# The role of C1q in recognition of apoptotic epithelial cells and inflammatory cytokine production by phagocytes during *Helicobacter pylori* infection

**DOI:** 10.1186/s12950-015-0098-8

**Published:** 2015-09-08

**Authors:** Sarah Fox, Kieran A. Ryan, Alice H. Berger, Katie Petro, Soumita Das, Sheila E. Crowe, Peter B. Ernst

**Affiliations:** Department of Pathology, University of California, La Jolla, San Diego, CA USA; Division of Gastroenterology and Hepatology, University of Virginia, Charlottesville, VA USA; National University Ireland, Galway, Ireland; Broad Institute of MIT and Harvard, Boston, MA USA; Athersys, Inc, Cleveland, OH USA

**Keywords:** Macrophage, Epithelial cell, H. pylori, C1q, Binding, Cytokines

## Abstract

**Background:**

Gastric epithelial cells (GECs) undergo apoptosis during *H. pylori* infection and phagocytes within the mucosa engulf these cells. The recognition and clearance of apoptotic cells is a multifactorial process, enhanced by the presence of various bridging molecules and opsonins which are abundant in serum. However, it is not clear how recognition or clearance may differ in the context of *H. pylori* infection induced apoptosis. In addition, efferocytosis of sterile apoptotic cells is known to confer anti-inflammatory properties in the engulfing phagocyte, however it is unknown if this is maintained when phagocytes encounter *H. pylori-*infected cells. Thus, the ability of macrophages to bind and engulf gastric epithelial cells rendered apoptotic by *H. pylori* infection and the association of these interactions to the modulation of phagocyte inflammatory responses was investigated in the absence and presence of serum with a particular focus on the role of serum protein C1q.

**Methods:**

Control (uninfected) or *H. pylori*-infected AGS cells were co-cultured with THP-1 macrophages in the presence or absence of serum or serum free conditions + C1q protein (40–80 μg/mL). Binding of AGS cells to THP-1 macrophages was assessed by microscopy and cytokine (IL-6 and TNF-α) release from LPS stimulated THP-1 macrophages was quantified by ELISA.

**Results:**

We show that macrophages bound preferentially to cells undergoing apoptosis subsequent to infection with *H. pylori*. Binding of apoptotic AGS to THP-1 macrophages was significantly inhibited when studied in the absence of serum and reconstitution of serum-free medium with purified human C1q restored binding of macrophages to apoptotic cells. Co-culture of sterile apoptotic and *H. pylori-*infected AGS cells both attenuated LPS-stimulated cytokine production by THP-1 macrophages. Further, direct treatment of THP-1 macrophages with C1q attenuated LPS stimulated TNF-α production.

**Conclusions:**

These studies suggest that C1q opsonizes GECs rendered apoptotic by *H. pylori*. No differences existed in the ability of infected or sterile apoptotic cells to attenuate macrophage cytokine production, however, there may be a direct role for C1q in modulating macrophage inflammatory cytokine production to infectious stimuli.

**Electronic supplementary material:**

The online version of this article (doi:10.1186/s12950-015-0098-8) contains supplementary material, which is available to authorized users.

## Background

*Helicobacter pylori*, a Gram-negative spiral bacterium, is one of the most common infections worldwide [1–3]. It colonizes the stomach of humans, usually in infancy [4], and has been implicated in a number of diseases including gastritis, gastroduodenal ulceration and gastric cancer [3, 5–7]. Approximately 20 % of individuals infected with *H. pylori* develop overt disease symptoms, the severity of which is extremely variable. Many groups have focused on identifying *H. pylori* virulence factors that may contribute to disease severity and a number of candidates have been proposed, including the vacuolating cytotoxin VacA [8, 9] and the cytotoxin-associated gene product CagA [9–11]. However, disease progression appears to be multi-factorial in that it also depends on a number of host factors including the immune and inflammatory response [12–15]. This includes the secretion of cytokines by the gastric epithelium [16–18] or mucosal antigen presenting cells [19], increased recruitment of cells such as neutrophils, a robust IgA response [20, 21] and T cell polarization [22–25]. Ultimately, this chronic, active inflammatory response contributes to tissue damage and the subsequent pathogenesis of gastroduodenal disease.

Despite the impressive advances to date, much remains to be learned about the host responses that regulate the magnitude of the inflammation and tissue damage. One mechanism that impacts inflammation involves phagocytes and their interaction and uptake of apoptotic cells, which attenuates phagocyte inflammatory responses, leading to termination of inflammation and initiation of tissue repair [26, 27]. After encountering apoptotic cells, phagocytes produce less pro-inflammatory mediators and increase their expression of anti-inflammatory, pro-resolution factors such as IL-10 and TGF-β1 [28–30]. The clearance of apoptotic cells is influenced by many soluble factors and receptor interactions. C1q, the initiating protein of the complement cascade, has been shown to be an important opsonin of apoptotic cells, enhancing their recognition and removal by phagocytes [31–33]. The importance of C1q is underscored in the autoimmune disease, systemic lupus erythematosus (SLE) where patients have a deficiency in C1q, from which the pathology has been linked to failed clearance of apoptotic cells [34].

In the present study, we examined the processes whereby gastric epithelial cells (AGS cells) are recognized by macrophages (THP-1 macrophages) in response to apoptosis induced by *H. pylori* infection. The attenuation of inflammatory responses by phagocytes following co-culture with sterile and infected apoptotic epithelial cells was also compared. As the complement protein C1q is one of several factors involved in the clearance of apoptotic cells [31, 32] and in light of recent reports of a direct role for C1q in modulation of macrophage inflammatory responses [35–39], we investigated the role of C1q in the interaction of infected gastric epithelial cells with phagocytes as well as its role in modulation of phagocyte cytokine responses.

## Methods

### Bacterial culture, cell lines and reagents

*Helicobacter pylori* strain 26695 was maintained routinely on blood agar plates containing 5 % horse blood (BD Pharmingen, San Jose, CA) at 37 °C in 10 % CO_2_. Prior to infection of cell cultures, bacteria were amplified in Brucella broth containing 10 % heat-inactivated FBS for 18 h. The AGS human gastric epithelial cell line and the THP-1 monocyte-like cell line were obtained from ATCC (Rockville, MD). The gastric epithelial cell line, AGS cells, were maintained in Dulbecco’s Modified Eagle’s Medium (DMEM) and THP-1 in RPMI 1640 (Gibco, NY), both containing 10 % heat-inactivated fetal bovine serum (FBS, Sigma, St. Louis, MO), at 37^ o^C in 5 % CO_2_. THP-1 cells were differentiated into a macrophage-like phenotype by treating with 600 nM phorbol myristate acetate (PMA; Sigma, St Lois, MO) for 3 days. Apoptosis was induced in AGS cells by infection with *H. pylori* as previously described [19] or by treatment with 3 μM camptothecin (Sigma, St. Louis, MO) for 24 h which proved a non-infectious means to induce apoptosis. Stimulation of apoptosis was carried out in the presence of 10 % FBS (heat-inactivated) medium after which the cells were washed twice in PBS (300 × *g*; 5 min) before being resuspended in the appropriate media prior to macrophage co-culture. The concentration of camptothecin or *H. pylori* used has previously been determined to be an optimal concentration to induce apoptosis in epithelial cells. Purified human C1q protein was purchased from Quidel (San Diego, CA).

### Preparation of human monocyte-derived macrophages

Human monocytes were isolated from blood drawn from healthy volunteers using a well-established technique involving dextran sedimentation followed by Percoll gradient separation [26]. Mononuclear cells were suspended in DMEM supplemented with 10 % autologous serum at 1 × 10^6^ cells/ml, and then 1 ml of the suspension was added to individual wells on a 24-well plate. The plate was incubated for 1 h at 37 °C and 5 % CO_2_, after which time non-adherent cells were removed by washing. Maturation of the mononuclear cells into macrophages was induced by culturing for 5–7 days in DMEM with 10 % autologous serum. All procedures using human blood were approved in advance by the Institutional Review Board of the University of Virginia and University of California, San Diego.

### Evaluation of phagocyte-target interactions by microscopy

Mature THP-1-derived macrophages were gently scraped and seeded onto an 8-well chamber slide (NUNC, Naperville, IL) at a density of 3 × 10^4^ and grown overnight. The same day, epithelial cells were infected with *H. pylori* strain 26695 at a MOI of 300:1 or treated with camptothecin (3 μM). The following day, the macrophages were incubated with 2.5 μg/ml of the cytoplasmic dye CMFDA (Molecular Probes, Eugene, OR) for 1 h at 37 °C and 5 % CO_2_. Epithelial cells were trypsinized and stained in a similar manner with 2.5 μg/ml of the dye SNARF® (Molecular Probes). Both dyes freely cross the cytoplasmic membrane, where they are modified by intracellular esterases to yield a fluorescent product, which cannot cross the cell membrane. Both sets of cells were washed three times, and then apoptotic or control AGS cells were added to individual wells of the chamber slide containing macrophages at a ratio of 5:1 in media containing normal FCS that was not heat inactivated. Results with FCS were comparable to using human serum (data not shown). Interactions were allowed to proceed for 1 h at 37 °C and 5 % CO_2_, after which time the reaction was stopped with PBS containing 0.01 % sodium azide. The slide was washed three times with PBS, fixed with 2 % paraformaldehyde for 40 min at 37 °C, and then the plastic wells and silicon adhesive were removed and discarded. Finally the slide was mounted with a cover slip and allowed to dry. Recognition and binding of apoptotic epithelial cells by and macrophages were assessed by counting using an Axioscope fluorescent microscope (Zeiss, Thornwood, NY). For each condition, four randomly selected fields in replicate slides were photographed and the total number of green fluorescent cells (macrophages) and red fluorescent cells (AGS) were counted. The number of macrophages with one, two or three or more AGS attached was recorded.

### Evaluation of cytokine responses

THP-1 cells were differentiated into a macrophage-like phenotype by treating with 600 nM phorbol myristate acetate (PMA; Sigma, St Louis, MO) for 3 days in 48-well plates. Cell numbers in the wells were approximately 100,000 and were quantified prior to the cell co-culture assay by trypsinizing cells and counting in a heamocytometer. AGS cells were made apoptotic with either camptothecin treatment (3 μM) or with *H. pylori* infection (MOI 100) overnight in T75 cm flasks. AGS cells were approx. 80–90 % confluent before infection and cell numbers were counted from a spare, identically seeded flask to calculate the appropriate MOI for the infection. On the day of the co-culture, AGS cells were trypsinized, washed twice in PBS (300 × g for 5 min), counted, and resuspended in THP-1 media (RPMI + 10 % FBS+ 10 mM HEPES + pen strep) or X-vivo 10 media (+2 mM L-glut) at 3 times the THP-1 cell number per ml (phagocyte:target ratio 3:1). The supernatant was removed from the THP-1 macrophages and washed using PBS to remove serum traces before addition of 1 ml of the AGS cell suspension. Where appropriate, the THP-1 macrophages were pre-treated with 5 μg/ml cytochalasin D (Sigma, St Louis, MO) for 30 min prior to and throughout AGS co-culture. The co-culture was performed for 2 h at 37 °C after which, the AGS cells were washed away from the adherent THP-1 cell monolayer using cold PBS (1 ml; × 3 washes). Media was replaced +/− LPS 100 ng/ml (from *Salmonella*; Sigma, St Louis, MO) for 24 h after which, the supernatant was removed, centrifuged at 10,000 × *g* for 15 min and stored at −80 °C until used for specific ELISA for IL-6 or TNF-α (R&D Systems, Minneapolis, MN).

### Polymerase chain reaction

Total RNA was extracted according to the manufacturer’s instructions using the RNeasy kit (Qiagen, Valencia, CA), and yield estimated spectrophotometrically. RNA was reverse transcribed using the Superscript kit (Invitrogen, Carlsbad, CA) random hexamer protocol as per the manufacturer’s instructions. Amplification of gC1qR, cC1qR, C1qRp, and CD91 mRNA was performed using TaqMan® pre-designed primers from Applied Biosystems (Foster City, CA). Primers were diluted 1:20 in TaqMan® Universal PCR Master Mix and water. Real-time PCR analysis was performed on a SmartCycler® (Cepheid, Sunnydale, CA) at 50 °C for 2 min, 95 °C for 10 min, 40 cycles of 95 °C for 15 s and 60 °C for 1 min.

### Flow cytometry

Mouse anti-human phycoerythrin (PE)-conjugated monoclonal antibodies (mAb) to C1qRp (product #551087), FITC-conjugated mAb to CD91 (product #550496), purified anti-calreticulin (product #612137), a FITC-rat-anti-mIgG_1_ secondary antibody, (product #562026) were obtained from BD Biosciences (San Diego, CA). A purified mouse anti-human mAb to gC1qR was a generous gift from Dr. Young Hahn (University of Virginia). FITC-conjugated rabbit polyclonal anti-human C1q was purchased from Abcam (product # ab4223, Cambridge, MA). PE-mIgG_2b,κ_, (product #556656) PE-mIgG_1,κ_ (product #551436), FITC- mIgG_1,κ_ (product #554679), purified mIgG_1,κ_ (product #349040) (BD Biosciences, San Diego CA) and FITC-Rabbit IgG (product #F0382), (Sigma, St Louis, MO) served as the isotype controls.

The assessment of apoptosis was performed using the Annexin V-FITC/Propidium iodide (PI) staining kit (R&D, Minneapolis, MN) as per manufacturer’s instructions. Cells were analyzed on a FACScan flow cytometer and analysed using FlowJo software (Treestar, Ashland, OR).

For complement receptor experiments, cells were suspended at 10^7^/ml in PBS containing 1 % BSA and 0.01 % sodium azide. Antibodies were incubated with cells for 30 min on ice, washed twice in PBS containing 0.01 % sodium azide, and resuspended in 2 % PFA. When required, secondary labeling was performed as per primary labelling, following two washes in PBS with 0.01 % azide. Cells were analysed within 24 h on a BD FACSCalibur flow cytometer, and the data subsequently analysed using FlowJo software (Treestar, Ashland, OR).

### C1q opsonization

Cells were seeded and allowed to adhere for 24 h prior to treatment with *H. pylori* (MOI 300:1), camptothecin (3 μM) or PBS (control cells). All treatments were performed in medium containing heat-inactivated serum. After 24 h of treatment, supernatants were collected, cells were washed once with PBS and trypsinzed. Trypsinized cells were added to supernatants and the cells then washed once in PBS. Cells were resuspended in either serum-free (SF) medium, medium containing 10 % non-heat-inactivated serum, or SF medium with 40 μg/ml C1q. After 1 h, cells were washed twice in PBS and resuspended at 10^7^ per ml in PBS with 0.01 % sodium azide. FITC-Rabbit IgG or FITC-anti-C1q was added for 30 min on ice. Cells were subsequently washed twice in PBS with 0.01 % sodium azide and resuspended in 2 % PFA until flow cytometry was performed.

### C1q cytokine studies

THP-1 cells were seeded at 0.25 × 10^6^/ml in 48 well plates in the presence of 600 nM PMA for 3 days. On the day of the assay, the number of macrophages per well was approximately 100,000 and was verified using a hemocytometer. The cells were washed with PBS and re-suspended in X-vivo 10 medium (+2 mM L-glut). C1q protein was added to the cells at 0–80 μg/ml for 30 min prior to the addition of LPS (100 ng/ml) or *H. pylori* (MOI 100). Unstimulated controls were run in parallel. After 24 h of treatment, supernatants were collected, centrifuged at 10,000 ×g for 10 min and stored at −80 °C until assessed for cytokine (IL-6 and TNF-α) concentration using specific ELISAs (R&D Systems, Minneapolis, MN).

### Statistics

Results are expressed as the mean ± SEM. Results were compared using two-tailed Student’s *t*-test, or one-way ANOVA with Tukey’s post-hoc correction when indicated. Statistical significance and p values are indicated in the figures.

## Results

### *H. pylori* infected gastric epithelial cells exhibit increased binding to macrophages

The engulfment of apoptotic cells involves recognition of the apoptotic target; binding of the target to the phagocyte and subsequent internalization into the cell. To determine whether infection of epithelial cells with *H. pylori* affected the ability of AGS cells to be recognized and bound to macrophages, control (uninfected) or infected AGS cells were incubated for 1 h with THP-1-derived macrophages. Evidence of binding was based on the number of epithelial cells (0, 1, 2 and ≥3 or more) associated with the phagocytes.

**Fig. 1:**
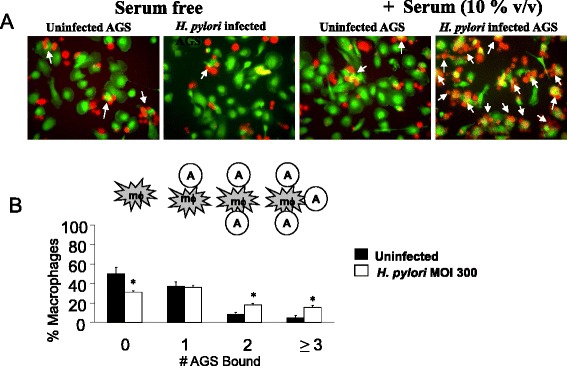
Infection of AGS cells with *H. pylori* increases interactions with macrophages and these interactions are facilitated by C1q. **a** The association of macrophages (*green*) and AGS cells (*red*) is shown in the absence and presence of serum, with either uninfected AGS cells or AGS cells that had been infected with *H. pylori* (MOI 300) for 24 h. Macrophages with aggregates of attached AGS cells are marked with a white arrow. **b** THP-1 macrophages were incubated for 1 h with either uninfected AGS (black) or *H. pylori*-infected (MOI 300) AGS (white) cells after which time the slide was washed three times and the number of bound AGS cells was counted in four random fields. Data shown is the average of *n* = 5–7 independent experiments. * overall *P* value of < 0.05

Figure 1a demonstrates representative images that were scored while Fig. 1b shows summary data collected from multiple, randomly selected fields from several slides for each condition. Based on these fluorescent images (Fig. 1a), it is clear that the recognition and binding of AGS cells were enhanced markedly if they were infected previously with *H. pylori* in the serum containing conditions. We also note that interactions between macrophages and AGS cells were virtually abrogated in the absence of serum in both uninfected and *H. pylori* infected conditions.

The ability of the THP-1 cells to recognize and bind uninfected and *H. pylori* infected AGS cells were compared (Fig. 1b). There was no significant difference between the numbers of macrophages with a single AGS cell attached, whether or not the AGS cells had been infected. However, aggregates of three or more AGS cells were bound to macrophages at a higher frequency when the AGS cells had been infected previously with *H. pylori* compared with uninfected cells. Consequently, the number of macrophages with no AGS cells clearly associated was much higher in the uninfected group than the infected targets, and this was also statistically significant (50 vs. 31 % for THP-1-derived macrophages, *p* < 0.05).

### Binding of apoptotic gastric epithelial cells to phagocytes is dependent on serum proteins and can be mediated by C1q

**Fig. 2:**
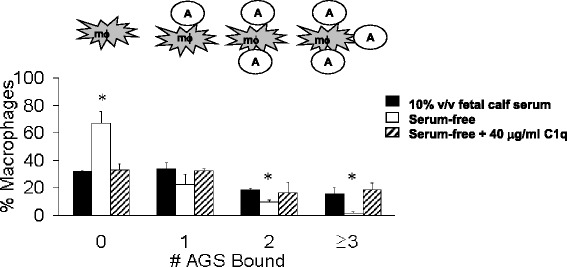
Reconstitution of C1q restores binding of apoptotic AGS cells to THP-1 cells in the absence of serum. THP-1 cells were incubated for 1 h with *H. pylori*-infected (MOI 300) AGS cells in the presence of 10 % v/v non-heat inactivated fetal calf serum (*black*), in serum-free media (*white*), or serum-free + 40 μg/ml C1q (diagonal bars), after which time the slide was washed three times and the number of bound AGS cells was counted in four random fields. Data shown is the average of *n* = 5 independent experiments. * overall *P* value of < 0.05

Recognition and binding of apoptotic AGS cells by macrophages were greatly reduced in the absence of serum (Fig. 2). Since complement proteins have been described to be important in apoptotic cell engulfment, we supplemented serum-free medium with 40 μg/ml of the complement component C1q. The addition of C1q was sufficient to increase the number of *H. pylori*-infected AGS cells in aggregates of three or more cells associated with macrophages (19 %) to levels similar to that seen in the presence of serum that hadn’t been heat-inactivated (15 %). The increase in macrophages binding epithelial targets resulted in a concomitant decrease in the number of macrophages with no associated AGS cells observed under serum-free vs. C1q-supplemented conditions (68 vs. 33 %, *p* < 0.05).

### Induction of Apoptosis of AGS cells by camptothecin and *H. pylori*

**Fig. 3:**
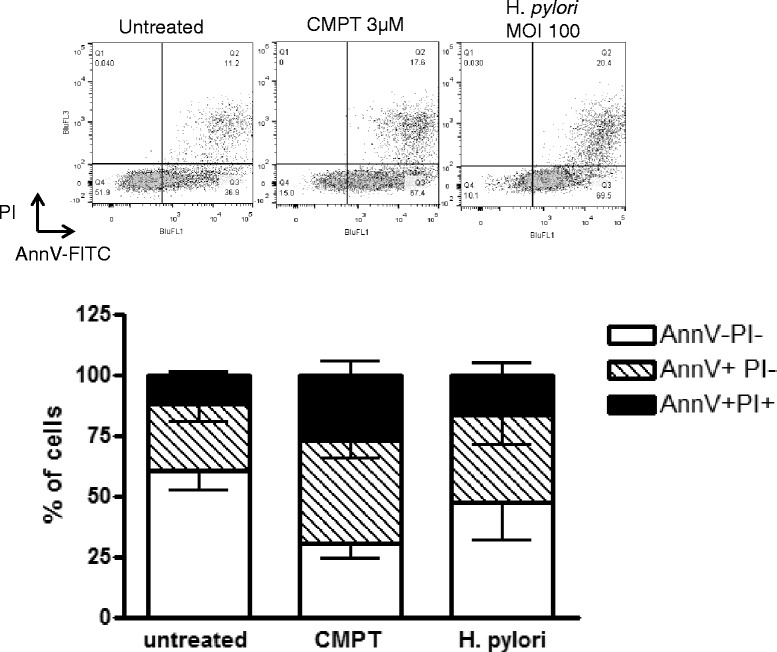
Induction of apoptosis in AGS cells. Induction of apoptosis in AGS cells by camptothecin (3 μM; 24 h) and *H. pylori* (MOI 100; 24 h). The top panel shows representative dot plots of AGS cells stained with Annexin V-FITC and PI in control untreated cells (*left*), following camptothecin treatment (*middle*) and *H. pylori* treatments (*right*). The bottom graph shows cumulative data from four independent experiments. AnnV-PI- are considered healthy, AnnV + PI- are considered to be apoptotic and AnnV + PI+ are cells which have progressed to secondary necrosis

We confirmed the levels of apoptosis in AGS cells by staining the cells with Annexin V and PI. We compare the induction of apoptosis of *H. pylori* with a common, sterile pro-apoptotic stimuli, camptothecin (3 μM). We demonstrate that *H. pylori* treatment increases the population of Annexin V positive cells compared to untreated cells. The induction of apoptosis by *H. pylori* was more modest than camptothecin, however we observe an increase in apoptosis that is consistent with previously published observations (Fig. 3) [40–43].

### C1q interacts with both the apoptotic targets and the phagocyte

**Fig. 4:**
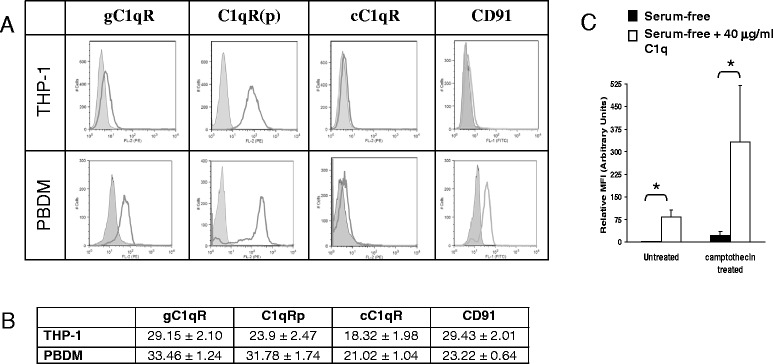
Complement receptors are expressed on THP-1-derived macrophages and PBDM’s. **a** THP-1 cells or PBDM’s were incubated with antibodies to indicated C1q receptors or matched isotype controls for 30 min, washed, fixed, and analyzed by flow cytometry. **b** RNA from THP-1 cells or PBDM’s was isolated and mRNA for the indicated C1q receptors was amplified using Real-Time PCR. Values in the table represent mean critical threshold ± standard deviation. Data shown is the average of at least three independent experiments. **c** C1q binds to apoptotic AGS cells. AGS cells were treated with 3 μM camptothecin for 24 h, trypsinized, washed and then incubated in serum-free media (black) or serum-free + 40 μg/ml C1q (white) for 1 h. Cells were then incubated with a C1q-FITC antibody for 30 min and C1q binding was measured using flow cytometry. Data shown is the average of three independent experiments. * overall *P* value of <0.05

Since the addition of C1q to serum-free medium restored binding of *H. pylori*-infected AGS cells to macrophages, it was possible that C1q served as a bridge between the apoptotic cell and the phagocyte. Phagocytes are known to bind C1q by means of different receptors [44, 45] so the expression of C1q receptors (gC1qR, C1qRp, and cC1qR or calreticulin) and an associated protein (CD91) were assessed. As shown in Fig. 4, PBDM’s expressed high levels of surface gC1qR and C1qRp. They expressed more modest levels of CD91 and cC1qR. Although THP-1 cells expressed high levels of C1qRp and modest levels of gC1qR, we were unable to detect surface cC1qR and CD91 on these cells (Fig. 4a, b).

It has been reported that the globular domain of C1q can bind the membrane blebs on apoptotic cells [31, 46]. In support of this notion, we set out to evaluate this in our model using apoptotic AGS. To rule out any nonspecific binding of C1q to *H. pylori,* camptothecin was used to induce apoptosis in AGS cells and then the cells were incubated in either serum-free medium or serum-free medium supplemented with 40 μg/ml C1q. The amount of C1q bound to the apoptotic cells was then assessed using a FITC conjugated antibody against C1q and analysed using flow cytometry. C1q was not detected in significant levels on the surface of AGS cells in the absence of serum (Fig. 4c) showing that although we induce apoptosis in serum containing conditions, this is not adequate to opsonize the cells. However, when AGS cells were incubated in medium containing additional C1q, high levels of C1q were shown to bind preferentially to the AGS cells that were stimulated to undergo apoptosis. With the data above, these observations support the notion that C1q is sufficient to bind apoptotic cells and allow detection by phagocytes expressing complement receptors which can collaborate in the clearance of the dead cells.

### Sterile apoptotic and *H. pylori* infected AGS cells attenuate THP-1 LPS-induced pro-inflammatory cytokine production

**Fig. 5:**
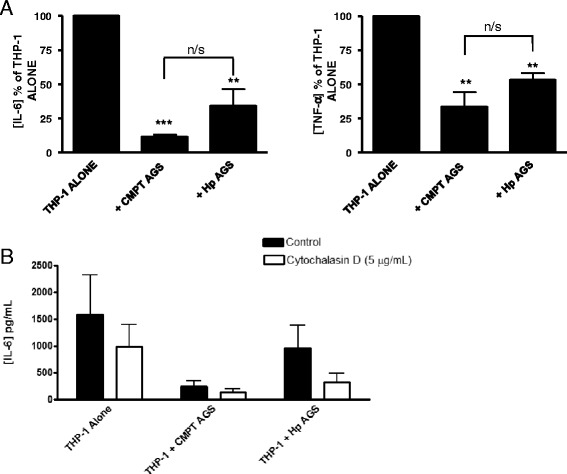
Apoptotic AGS co-culture attenuates THP-1 macrophage production of inflammatory cytokines irrespective of the method of apoptosis induction. **a** AGS made apoptotic with camptothecin (CMPT; 3 μM) or with *H. pylori* (MOI 100) for 24 h were co-incubated with THP-1 macrophages for 2 h. Apoptotic AGS were washed off and the THP-1 macrophages were replaced with RPMI+ 10 % FBS + 10 mM HEPES + 100 ng/mL LPS. Supernatants were collected after 24 h of LPS stimulation and were stored at −80 °C until analyzed for IL-6 and TNF-α concentrations using specific ELISA. **b** shows that attenuation of LPS-stimulated (100 ng/mL) IL-6 release was independent of internalization as the presence of cytochalasin D (CytD; 5 μg/mL) treatment did not reverse the attenuation of IL-6 observed following CMPT or H. *pylori* apoptotic AGS co-culture. Data shown is the average of three-four independent experiments. * *P* value of < 0.05, ** *P* value of < 0.01

The immunomodulatory properties of apoptotic AGS cells were investigated using an in vitro co-culture assay. Figure 5a shows that the co-culture of sterile (camptothecin induced) apoptotic, and *H. pylori* infected AGS cells significantly attenuated LPS-stimulated production of IL-6 and TNF-α by THP-1 macrophages compared to THP-1 cells that did not contact AGS cells (THP-1 alone). Both treatment groups attenuated LPS induced cytokine production although sterile apoptotic cells appear to be more effective, however statistical significance between the groups was never reached. It was noted that internalization of the apoptotic targets was not required for the co-culture associated attenuation of cytokine production as treatment of the co-cultures with cytochalasin D had no effect (Fig. 5b).

**Fig. 6:**
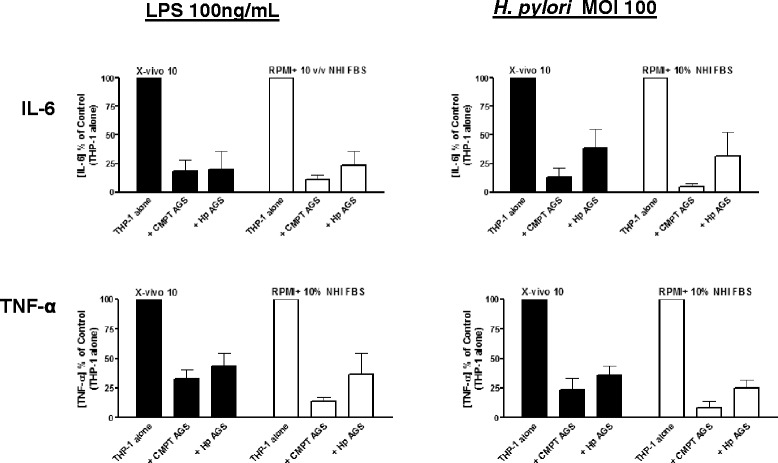
Attenuation of IL-6 and TNF-α production following apoptotic AGS co-incubation is not dependent on serum factors. Attenuation of IL-6 and TNF-α production following apoptotic AGS co-incubation is not dependent on serum factors. THP-1 macrophages were resuspended in serum free media (X-vivo10) or serum containing media (RPMI + 10 % v/v Non-Heat Inactivated FBS) and co-cultured with camptothecin (3 μM; 24 h) induced apoptotic AGS (CMPT AGS) or *H. pylori* (MOI 100; 24 h) infected AGS (Hp AGS) which were in the appropriate medium (THP-1: AGS ratio of 1:3). After 2 h co-incubation, apoptotic AGS were washed off with PBS. THP-1 macrophages were resuspended in the appropriate media (X-vivo 10 or RPMI + 10 % Non-heat inactivated FBS) and stimulated with LPS (100 ng/mL) or *H. pylori* (26695, MOI 100) for 24 h before collection of supernatants. Supernatants were centrifuged 10,000 × *g* for 10 min and stored at −80 °C until analyzed for IL-6 and TNF- α using specific ELISA. Data are shown as percent Control (THP-1 alone) of the relevant media condition from three independent experiments. Data comparisons between treatments performed in X-vivo 10 and RPMI + 10 % NHI FBS conditions were not significantly different

The attenuation of cytokine production was independent of the presence of serum factors as co-cultures performed in X-vivo 10 medium (serum free) were able to attenuate cytokine production to a similar degree as those observed in 10 % v/v Non-heat inactivated (NHI) FBS conditions (Fig. 6). This held true for both LPS and *H. pylori* stimulated conditions.

### C1q protein attenuates THP-1 macrophage production of cytokines in response to LPS and *H. pylori* stimulation

**Fig. 7:**
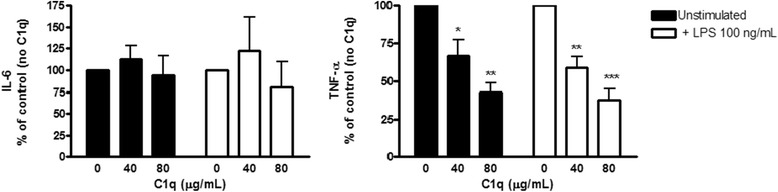
C1q protein inhibits THP-1 macrophage inflammatory cytokine release. THP-1 macrophages were washed and resuspended in serum free medium (Xvivo 10 + L-glut) and pretreated with C1q (0–80 μg/ml) for 30 min before stimulation with LPS (100 ng/ml) for 24 h. Unstimulated controls were run in parallel. Supernatants were collected and TNF-α and IL-6 were measured by specific ELISA. Data shown is the average of *n* = 3–4 (IL-6 *n* = 3, TNF-α *n* = 4) independent experiments. * *P* value of <0.05, ** *P* value of <0.01, *** *P* value of <0.001

The direct effect of C1q protein on LPS and *H. pylori* stimulated (MOI 100) cytokine production by THP-1 macrophages was assessed (Fig. 7 and Additional file 1: Figure S1). The assays were performed in serum free medium to assess the effect of C1q in the absence of other complement or serum factors. We report a significant dose-dependent attenuation of THP-1 macrophage production of TNF-α under baseline (unstimulated) and LPS-stimulated conditions. Preliminary experiments evaluating cytokines following *H. pylori* stimulation (Additional file 1: Figure S1) compare the effects of C1q on IL-6 and TNF-α production.

## Discussion

Engulfment of apoptotic cells is an important process for tissue remodelling and homeostasis. This response entails several steps including: recruitment of phagocytes; recognition of dead cells; binding of the corpse to a phagocyte; internalization of the target; target degradation and finally, modification of phagocyte cell function. Engulfment of apoptotic epithelial cells by macrophages or dendritic cells has been observed in the intestinal lamina propria [47–49], the stomach [50] and in the lung [51]. Furthermore, epithelial cell apoptosis is highly inducible by infections [41, 52–54] and in response to inflammation [12, 55]. The engulfment of apoptotic cells does not only remove cells to prevent release of toxic cell contents, but it is now widely accepted that apoptotic cells promote an anti-inflammatory phenotype in the engulfing phagocyte, thereby limiting inflammation [26, 27]. The current report shows that C1q opsonizes apoptotic AGS cells and also confers immunomodulatory properties to the phagocyte. Our model used the human THP-1 cell line that is differentiated into macrophage like cells using PMA. Previous studies using peripheral blood derived macrophages (PBDM) have been published by our group, which show comparable levels of AGS cell binding following *H. pylori* infection [56]. Furthermore, our previous work also show attenuation of PBDM cytokine responses following apoptotic AGS co-culture and are in concordance with what we observe with THP-1 macrophages. Thus, our use of the THP-1 cell line as a model macrophage is appropriate and representative of other human macrophage responses.

In healthy cells, nearly all of the phosphatidylserine (PS) is confined to the inner aspect of the plasma membrane and exposure of PS on the outer leaflet marks the cells as apoptotic. As discussed elsewhere [57] many receptors and adaptor molecules have been implicated in the recognition and clearance of apoptotic cells including collectin receptors, calreticulin/CD91, Fcγ receptors, c-Mer, integrins such as αvβ3, scavenger receptors thrombospondin–CD36 and phosphatidylserine receptors like BAI1 and TIM4. Some of the recognition structures that bind to PS trigger a signalling response leading to engulfment (i.e., BAI1 [58]) while others, such as Tim4, perform an accessory function that facilitates the PS-dependent uptake of the target [59, 60]. It has been reported that complement proteins also bind to apoptotic cells, marking them for quick removal, and a large panel of different complement proteins are involved, including C1q, mannose-binding lectin and C3bi [61, 62]. The present study revealed that the interactions between *H. pylori*-infected cells and macrophages were mediated by a serum component and that C1q was sufficient to restore binding of *H. pylori*-infected AGS cells to macrophages. Importantly, C1q also conferred an anti-inflammatory effect on macrophages stimulated with LPS or *H. pylori.* The concentrations of C1q used in our experiments (40–80 μg/mL) are well within the normal range found in plasma (56–276 μg/mL). In relation to *H. pylori* infection, Berstad and colleagues [63, 64] have detected the terminal complement complex (TCC) in the gastric mucosa of subjects infected with *H. pylori* showing that complement activation occurs during infection, which could regulate the interaction between APCs and apoptotic cells as well as regulate phagocyte reactivity to infection.

It has been reported that C1q can bind via its globular head to PS exposed on the surface membrane blebs of apoptotic cells [31, 32, 46]. Indeed, induction of apoptosis in AGS cells with camptothecin allowed C1q to bind the target suggesting that the interaction was through the recognition of the dead cell and no bacterial component is required for the binding by C1q to occur. The fact that THP-1 cells expressed multiple receptors for C1q (gC1qR and C1qRp), supports the feasibility of the interaction. We analyzed the expression of peripheral blood derived macrophages (PBDMs) and showed these cells also express receptors for C1q. Using siRNA approaches to inhibit individual receptor expression were successful based on assays to measure gene expression. However, they failed to significantly block the C1q-mediated binding of the apoptotic cell (data not shown). This level of redundancy is common in binding and engulfment studies due to the presence of multiple receptors and mechanisms that phagocytes utilize to clear a wide array of different targets.

Several studies have demonstrated an effect of C1q on cytokine production by monocytes, macrophages and DCs during apoptotic cell uptake [35–37]. The modulation of cytokines by C1q was variable depending on which cytokines and APC were being studied. In general, a reduction in pro-inflammatory cytokines, IL-1β and IL-1α were reported together with an increase in IL-10, IL-6 and MCP-1. In the current study, apoptotic AGS cells attenuated production of LPS-stimulated cytokines, IL-6 and TNF-α independent of target internalization or the presence of serum. Moreover, the attenuation of cytokine responses occurred regardless of the method used to induce apoptosis. It is important to note that infected cell co-cultures did not confer a net pro-inflammatory effect compared to macrophages alone. This is intriguing as there are reports of other bacterial species which can use apoptotic targets as a type of Trojan horse to invade phagocytes in order to disseminate to locations remote from the point of entry [65, 66]. Current work by our lab and others has noted the redundancy of apoptotic cell internalization for attenuated macrophage responses [56, 67]. In these studies, it has been shown that conditioned medium from apoptotic cells is insufficient to confer anti-inflammatory effects and that contact of the macrophage and apoptotic cells is required. Furthermore, we have previously shown that binding of PS residues is also not required for the attenuation response to occur [56]. The receptors responsible for altering macrophage reactivity following apoptotic cell contact are yet to be elucidated.

No serum components were required for the apoptotic cell-induced attenuation of cytokine responses and additional C1q during co-culture did not enhance this effect (data not shown). However, C1q protein incubated directly with THP-1 macrophages in the absence of apoptotic cells did have a significant effect, demonstrating a dose-dependent effect of C1q on LPS-stimulated TNF-α. The attenuation of TNF-α was noteworthy as this suggests a role for C1q in control of host responses to infection. We observed a similar trend in attenuation of baseline (unstimulated) production of TNF-α which may also indicate that C1q could be involved in controlling the general stress response in macrophages induced, in this instance, by serum starvation. Although the mechanism of the attenuation of cytokines by C1q remains elusive in our model, a recent publication by Galvan et al. has identified activation of AMPK as a potential candidate pathway which is triggered by C1q to induce modulation of immune reactivity in murine macrophages [68].

We show that *H. pylori*-infected AGS gastric epithelial cells bind to macrophages and that this response is facilitated by the C1q component of complement. It has been confirmed that these apoptotic cells are internalized by macrophages [56] although no additional enhancement of this process was observed with C1q in our hands (data not shown). As mentioned previously, the reported degree of receptor redundancy in the engulfment process makes it difficult to assess the importance of one factor [69]. Other investigations have shown that the maturation and type of APC can influence the C1q enhanced uptake of apoptotic cells [37]. They reported that monocytes but not monocyte-derived macrophages or DCs, enhanced their phagocytic capacity for apoptotic cells in the presence of C1q. However, enhancement of phagocytosis was observed in all APCs when C1q was added in the presence of serum with active complement. These differences in assay design may explain the lack of enhancement in internalization in our hands, as we only added C1q to serum free conditions to discriminate from the involvement of other serum factors. It is also important to note the differences in the apoptotic target cells used between other studies and ours, as our epithelial cell targets are larger than the conventionally used T cell (Jurkat) targets, and therefore may be more difficult to internalize.

Epithelial cell apoptosis occurs frequently in normal physiology, where the cells are replaced from progenitor cells located at the base of the crypts. [41, 53, 54]. Apoptotic epithelial cells are shed via extrusion, however at least some of these cells are cleared by phagocytes and neighbouring epithelial cells [70–72]. In this manner, the internalization of epithelial cells provides another means by which the host can sample the environment for antigens associated with damage or infection [73]. It has also been reported that apoptotic epithelial cells can influence inflammation [72, 74]. Juncadella et al. demonstrated that apoptotic bronchial epithelial cells can be engulfed by other epithelial cells and that these interactions are important in the control of murine airway inflammation [74]. Relevant to *H. pylori,* a recent study has shown that infection by *H. pylori* inhibits the phagocytosis of apoptotic gastric epithelial cells [75]. Inhibition of clearance and the concomitant increase in the release of necrotic cell contents would trigger a heightened inflammatory response, which would likely inhibit the anti-inflammatory effect conferred by apoptotic cells. Furthermore, decreased phagocytosis would result in the reduced sampling of infected cells, which could lead to reduced antigen presentation and less pathogen specific immune cell attack. These mechanisms may provide an explanation of how a predominantly luminal infection triggers such a potent immune reaction. Future studies should focus on using the more relevant tissue specific macrophages as it is becoming apparent that these cells can respond very differently to microbial insult [76].

## Conclusions

We report that human epithelial cells rendered apoptotic by infection are recognized and bound by phagocytes. This response entails a role for complement proteins, with C1q being a likely candidate. The co-culture of sterile (camptothecin induced) apoptotic and *H. pylori* infected AGS cells with macrophages resulted in an attenuated phagocyte cytokine response to LPS compared to phagocytes that did not contact apoptotic AGS cells and the attenuation was independent of the method of apoptosis induction and internalization of the apoptotic cell. The attenuation of LPS and *H. pylori* induced cytokine responses following apoptotic cell co-culture was independent of the presence of serum components, as the attenuation was observed in serum free conditions. Although serum components were not required for the effect observed following apoptotic cell co-culture, we show that incubation of C1q protein itself with macrophages was able to dose-dependently diminish LPS-stimulated production of TNF-α, thus, indicating that this protein could be involved in directly controlling the host response to infection. The outcome of this process on the regulation of host responses in the gastric mucosa remains the subject for future study.

## Additional file

Additional file 1: Figure S1.
**C1q protein inhibits THP-1 macrophage** ***H. pylori*** **stimulated inflammatory cytokine release.** THP-1 macrophages were washed and resuspended in serum free medium (Xvivo 10 + L-glut) and pretreated with C1q (0–80 μg/ml) for 30 min before stimulation with *H. pylori* (MOI 100) for 24 h. Supernatants were collected and TNF-α and IL-6 were measured by specific ELISA. The average and individual values of 2 replicates from *n* = 2 independent experiments are shown. (PPTX 55 kb)

## References

[1] World Gastroenterology Organisation Global Guideline (2011). Helicobacter pylori in developing countries. J Clin Gastroenterol.

[2] Hunt RH, Xiao SD, Megraud F, Leon-Barua R, Bazzoli F, van der Merwe S, Vaz Coelho LG, Fock M, Fedail S, Cohen H (2011). Helicobacter pylori in developing countries. World Gastroenterology Organisation Global Guideline. J Gastrointestin Liver Dis.

[3] Polk DB, Peek RM (2010). Helicobacter pylori: gastric cancer and beyond. Nat Rev Cancer.

[4] Ernst PB, Gold BD (1999). Helicobacter pylori in childhood: new insights into the immunopathogenesis of gastric disease and implications for managing infection in children. J Pediatr Gastroenterol Nutr.

[5] Ernst PB, Peura DA, Crowe SE (2006). The translation of Helicobacter pylori basic research to patient care. Gastroenterology.

[6] Rad R, Prinz C, Schmid RM (2006). Helicobacter pylori and prognosis of gastric carcinoma. Lancet Oncol.

[7] Danese S, Papa A, Gasbarrini A, Ricci R, Maggiano N (2004). Helicobacter pylori eradication down-regulates matrix metalloproteinase-9 expression in chronic gastritis and gastric ulcer. Gastroenterology.

[8] Ghiara P, Marchetti M, Blaser MJ, Tummuru MK, Cover TL, Segal ED, Tompkins LS, Rappuoli R (1995). Role of the Helicobacter pylori virulence factors vacuolating cytotoxin, CagA, and urease in a mouse model of disease. Infect Immun.

[9] Blaser MJ (2012). Heterogeneity of Helicobacter pylori. Eur J Gastroenterol Hepatol.

[10] Blaser MJ (2005). The biology of cag in the Helicobacter pylori-human interaction. Gastroenterology.

[11] Yamaoka Y (2010). Mechanisms of disease: Helicobacter pylori virulence factors. Nat Rev Gastroenterol Hepatol.

[12] Ernst PB, Crowe SE, Reyes VE (1997). How does Helicobacter pylori cause mucosal damage? The inflammatory response. Gastroenterology.

[13] Persson C, Canedo P, Machado JC, El-Omar EM, Forman D (2011). Polymorphisms in inflammatory response genes and their association with gastric cancer: A HuGE systematic review and meta-analyses. Am J Epidemiol.

[14] Macarthur M, Hold GL, El-Omar EM (2004). Inflammation and Cancer II. Role of chronic inflammation and cytokine gene polymorphisms in the pathogenesis of gastrointestinal malignancy. Am J Physiol Gastrointest Liver Physiol.

[15] El-Omar E, Carrington M, Chow WH, McColl KEL, Bream JH, Young HA, Herrera J, Lissowska J, Yuan CC, Rothman N (2000). Interleukin-1 polymorphisms associated with increased risk of gastric cancer. Nature.

[16] Crabtree JE, Covacci A, Farmery SM, Xiang Z, Tompkins DS, Perry S, Lindley IJ, Rappuoli R (1995). Helicobacter pylori induced interleukin-8 expression in gastric epithelial cells is associated with CagA positive phenotype. J Clin Pathol.

[17] Crabtree JE, Shallcross TM, Heatley RV, Wyatt JI (1991). Mucosal tumour necrosis factor alpha and interleukin-6 in patients with Helicobacter pylori associated gastritis. Gut.

[18] Crowe SE, Alvarez L, Dytoc M, Hunt RH, Muller M, Sherman P, Patel J, Jin Y, Ernst PB (1995). Expression of interleukin 8 and CD54 by human gastric epithelium after Helicobacter pylori infection in vitro. Gastroenterology.

[19] Harris PR, Ernst PB, Kawabata S, Kiyono H, Graham MF, Smith PD (1998). Recombinant Helicobacter pylori urease activates primary mucosal macrophages. J Infect Dis.

[20] Wyatt JI, Rathbone BJ (1988). Immune response of the gastric mucosa to Campylobacter pylori. Scand J Gastroenterol Suppl.

[21] Hansson M, Hermansson M, Svensson H, Elfvin A, Hansson LE, Johnsson E, Sjoling A, Quiding-Jarbrink M (2008). CCL28 is increased in human Helicobacter pylori-induced gastritis and mediates recruitment of gastric immunoglobulin A-secreting cells. Infect Immun.

[22] Bimczok D, Grams JM, Stahl RD, Waites KB, Smythies LE, Smith PD (2011). Stromal regulation of human gastric dendritic cells restricts the Th1 response to Helicobacter pylori. Gastroenterology.

[23] Bamford KB, Fan XJ, Crowe SE, Leary JF, Gourley WK, Luthra GK, Brooks EG, Graham DY, Reyes VE, Ernst PB (1998). Lymphocytes in the human gastric mucosa during Helicobacter pylori have a T helper cell 1 phenotype. Gastroenterology.

[24] Luzza F, Parrello T, Monteleone G, Sebkova L, Romano M, Zarrilli R, Imeneo M, Pallone F (2000). Up-regulation of IL-17 is associated with bioactive IL-8 expression in Helicobacter pylori -infected human gastric mucosa. J Immunol.

[25] Harris PR, Wright SW, Serrano C, Riera F, Duarte I, Torres J, Pena A, Rollan A, Viviani P, Guiraldes E (2008). Helicobacter pylori gastritis in children is associated with a regulatory T-cell response. Gastroenterology.

[26] Savill J, Dransfield I, Gregory C, Haslett C (2002). A blast from the past: clearance of apoptotic cells regulates immune responses. Nat Rev Immunol.

[27] Savill J, Fadok V (2000). Corpse clearance defines the meaning of cell death. Nature.

[28] Huynh ML, Fadok VA, Henson PM (2002). Phosphatidylserine-dependent ingestion of apoptotic cells promotes TGF-beta1 secretion and the resolution of inflammation. J Clin Invest.

[29] Fadok VA, Bratton DL, Konowal A, Freed PW, Westcott JY, Henson PM (1998). Macrophages that have ingested apoptotic cells in vitro inhibit proinflammatory cytokine production through autocrine/paracrine mechanisms involving TGF-beta, PGE2, and PAF. J Clin Invest.

[30] Ferracini M, Rios FJ, Pecenin M, Jancar S (2013). Clearance of apoptotic cells by macrophages induces regulatory phenotype and involves stimulation of CD36 and platelet-activating factor receptor. Mediators Inflamm.

[31] Paidassi H, Tacnet-Delorme P, Garlatti V, Darnault C, Ghebrehiwet B, Gaboriaud C, Arlaud GJ, Frachet P (2008). C1q binds phosphatidylserine and likely acts as a multiligand-bridging molecule in apoptotic cell recognition. J Immunol.

[32] Ogden CA, de Cathelineau A, Hoffmann PR, Bratton D, Ghebrehiwet B, Fadok VA, Henson PM (2001). C1q and mannose binding lectin engagement of cell surface calreticulin and CD91 initiates macropinocytosis and uptake of apoptotic cells. J Exp Med.

[33] Vandivier RW, Ogden CA, Fadok VA, Hoffmann PR, Brown KK, Botto M, Walport MJ, Fisher JH, Henson PM, Greene KE (2002). Role of surfactant proteins A, D, and C1q in the clearance of apoptotic cells in vivo and in vitro: calreticulin and CD91 as a common collectin receptor complex. J Immunol.

[34] Gaipl US, Munoz LE, Grossmayer G, Lauber K, Franz S, Sarter K, Voll RE, Winkler T, Kuhn A, Kalden J (2007). Clearance deficiency and systemic lupus erythematosus (SLE). J Autoimmun.

[35] Benoit ME, Clarke EV, Morgado P, Fraser DA, Tenner AJ (2012). Complement protein C1q directs macrophage polarization and limits inflammasome activity during the uptake of apoptotic cells. J Immunol.

[36] Fraser DA, Bohlson SS, Jasinskiene N, Rawal N, Palmarini G, Ruiz S, Rochford R, Tenner AJ (2006). C1q and MBL, components of the innate immune system, influence monocyte cytokine expression. J Leukoc Biol.

[37] Fraser DA, Laust AK, Nelson EL, Tenner AJ (2009). C1q differentially modulates phagocytosis and cytokine responses during ingestion of apoptotic cells by human monocytes, macrophages, and dendritic cells. J Immunol.

[38] Verneret M, Tacnet-Delorme P, Osman R, Awad R, Grichine A, Kleman JP, Frachet P (2014). Relative contribution of c1q and apoptotic cell-surface calreticulin to macrophage phagocytosis. J Innate Immun.

[39] Clarke EV, Weist BM, Walsh CM, Tenner AJ (2015). Complement protein C1q bound to apoptotic cells suppresses human macrophage and dendritic cell-mediated Th17 and Th1 T cell subset proliferation. J Leukoc Biol.

[40] Jones NL, Day AS, Jennings HA, Sherman PM (1999). Helicobacter pylori induces gastric epithelial cell apoptosis in association with increased Fas receptor expression. Infect Immun.

[41] Moss SF, Calam J, Agarwal B, Wang S, Holt PR (1996). Induction of gastric epithelial apoptosis by Helicobacter pylori. Gut.

[42] Rudi J, Kuck D, Strand S, Von Herbay A, Mariani SM, Krammer PH, Galle PR, Stremmel W (1998). Involvement of the CD95 (APO-1/Fas) receptor and ligand system in Helicobacter pylori -induced gastric epithelial apoptosis. J Clin Invest.

[43] Fan XJ, Crowe SE, Behar S, Gunasena H, Ye G, Haeberle H, Van Houten N, Gourley WK, Ernst PB, Reyes VE (1998). The effect of class II MHC expression on adherence of Helicobacter pylori and induction of apoptosis in gastric epithelial cells: A mechanism for Th1 cell-mediated damage. J Exp Med.

[44] Ghebrehiwet B, Peerschke EI (2014). Purification of C1q receptors and functional analysis. Methods Mol Biol.

[45] Ghiran I, Tyagi SR, Klickstein LB, Nicholson-Weller A (2002). Expression and function of C1q receptors and C1q binding proteins at the cell surface. Immunobiology.

[46] Nauta AJ, Trouw LA, Daha MR, Tijsma O, Nieuwland R, Schwaeble WJ, Gingras AR, Mantovani A, Hack EC, Roos A (2002). Direct binding of C1q to apoptotic cells and cell blebs induces complement activation. Eur J Immunol.

[47] Iwanaga T, Han H, Adachi K, Fujita T (1993). A novel mechanism for disposing of effete epithelial cells in the small intestine of guinea pigs. Gastroenterology.

[48] Iwanaga T, Hoshi O, Han H, Takahashi-Iwanaga H, Uchiyama Y, Fujita T (1994). Lamina propria macrophages involved in cell death (apoptosis) of enterocytes in the small intestine of rats. Arch Histol Cytol.

[49] Shibahara T, Sato N, Waguri S, Iwanaga T, Nakahara A, Fukutomi H, Uchiyama Y (1995). The fate of effete epithelial cells at the villus tips of the human small intestine. Arch Histol Cytol.

[50] Karam SM (1993). Dynamics of epithelial cells in the corpus of the mouse stomach. IV. Bidirectional migration of parietal cells ending in their gradual degeneration and loss. Anat Rec.

[51] Borges VM, Vandivier RW, McPhillips KA, Kench JA, Morimoto K, Groshong SD, Richens TR, Graham BB, Muldrow AM, Van Heule L (2009). TNFalpha inhibits apoptotic cell clearance in the lung, exacerbating acute inflammation. Am J Physiol Lung Cell Mol Physiol.

[52] Fan X, Gunasena H, Cheng Z, Espejo R, Crowe SE, Ernst PB, Reyes VE (2000). Helicobacter pylori urease binds to class II MHC on gastric epithelial cells and induces their apoptosis. J Immunol.

[53] Jones NL, Shannon PT, Cutz E, Yeger H, Sherman PM (1997). Increase in proliferation and apoptosis of gastric epithelial cells early in the natural history of Helicobacter pylori infection. Am J Pathol.

[54] Wagner S, Beil W, Westermann J, Logan RP, Bock CT, Trautwein C, Bleck JS, Manns MP (1997). Regulation of gastric epithelial cell growth by Helicobacter pylori: offdence for a major role of apoptosis. Gastroenterology.

[55] Denning TL, Takaishi H, Crowe SE, Boldogh I, Jevnikar A, Ernst PB (2002). Oxidative stress induces the expression of Fas and Fas ligand and apoptosis in murine intestinal epithelial cells. Free Radic Biol Med.

[56] Das S, Sarkar A, Ryan KA, Fox S, Berger AH, Juncadella IJ, et al. Brain angiogenesis inhibitor 1 is expressed by gastric phagocytes during infection with Helicobacter pylori and mediates the recognition and engulfment of human apoptotic gastric epithelial cells. FASEB J. 2014. 10.1096/fj.13-243238 PMC3986834 24509909

[57] Bratton DL, Henson PM (2008). Apoptotic cell recognition: will the real phosphatidylserine receptor(s) please stand up?. Curr Biol.

[58] Park D, Tosello-Trampont AC, Elliott MR, Lu M, Haney LB, Ma Z, Klibanov AL, Mandell JW, Ravichandran KS (2007). BAI1 is an engulfment receptor for apoptotic cells upstream of the ELMO/Dock180/Rac module. Nature.

[59] Miyanishi M, Tada K, Koike M, Uchiyama Y, Kitamura T, Nagata S (2007). Identification of Tim4 as a phosphatidylserine receptor. Nature.

[60] Park D, Hochreiter-Hufford A, Ravichandran KS (2009). The phosphatidylserine receptor TIM-4 does not mediate direct signaling. Curr Biol.

[61] Bohlson SS, Fraser DA, Tenner AJ (2007). Complement proteins C1q and MBL are pattern recognition molecules that signal immediate and long-term protective immune functions. Mol Immunol.

[62] Hart SP, Smith JR, Dransfield I (2004). Phagocytosis of opsonized apoptotic cells: roles for ‘old-fashioned’ receptors for antibody and complement. Clin Exp Immunol.

[63] Berstad AE, Brandtzaeg P, Stave R, Halstensen TS (1997). Epithelium related deposition of activated complement in Helicobacter pylori associated gastritis. Gut.

[64] Berstad AE, Hogasen K, Bukholm G, Moran AP, Brandtzaeg P (2001). Complement activation directly induced by Helicobacter pylori. Gastroenterology.

[65] Laskay T, van Zandbergen G, Solbach W (2008). Neutrophil granulocytes as host cells and transport vehicles for intracellular pathogens: apoptosis as infection-promoting factor. Immunobiology.

[66] Nguyen L, Pieters J (2005). The Trojan horse: survival tactics of pathogenic mycobacteria in macrophages. Trends Cell Biol.

[67] Lucas M, Stuart LM, Zhang A, Hodivala-Dilke K, Febbraio M, Silverstein R, Savill J, Lacy-Hulbert A (2006). Requirements for apoptotic cell contact in regulation of macrophage responses. J Immunol.

[68] Galvan MD, Hulsebus H, Heitker T, Zeng E, Bohlson SS (2014). Complement Protein C1q and Adiponectin Stimulate Mer Tyrosine Kinase-Dependent Engulfment of Apoptotic Cells through a Shared Pathway. J Innate Immun.

[69] Mosser DM (1994). Receptors on phagocytic cells involved in microbial recognition. Immunol Ser.

[70] Hall PA, Coates PJ, Ansari B, Hopwood D (1994). Regulation of cell number in the mammalian gastrointestinal tract: the importance of apoptosis. J Cell Sci.

[71] Monks J, Smith-Steinhart C, Kruk ER, Fadok VA, Henson PM (2008). Epithelial cells remove apoptotic epithelial cells during post-lactation involution of the mouse mammary gland. Biol Reprod.

[72] Monks J, Rosner D, Geske FJ, Lehman L, Hanson L, Neville MC, Fadok VA (2005). Epithelial cells as phagocytes: apoptotic epithelial cells are engulfed by mammary alveolar epithelial cells and repress inflammatory mediator release. Cell Death Differ.

[73] Getts DR, McCarthy DP, Miller SD (2013). Exploiting apoptosis for therapeutic tolerance induction. J Immunol.

[74] Juncadella IJ, Kadl A, Sharma AK, Shim YM, Hochreiter-Hufford A, Borish L, Ravichandran KS (2013). Apoptotic cell clearance by bronchial epithelial cells critically influences airway inflammation. Nature.

[75] Bimczok D, Smythies LE, Waites KB, Grams JM, Stahl RD, Mannon PJ, Peter S, Wilcox CM, Harris PR, Das S (2013). Helicobacter pylori infection inhibits phagocyte clearance of apoptotic gastric epithelial cells. J Immunol.

[76] Smith PD, Smythies LE, Shen R, Greenwell-Wild T, Gliozzi M, Wahl SM (2011). Intestinal macrophages and response to microbial encroachment. Mucosal Immunol.

