# Supplementary figures and images for: The role of C1q in recognition of apoptotic epithelial cells and inflammatory cytokine production by phagocytes during Helicobacter pylori infection
Source: J Inflamm (Lond). 2015 Sep 8;12:51. doi: 10.1186/s12950-015-0098-8 (PMC4563842; doi:10.1186/s12950-015-0098-8)

## Slide 1
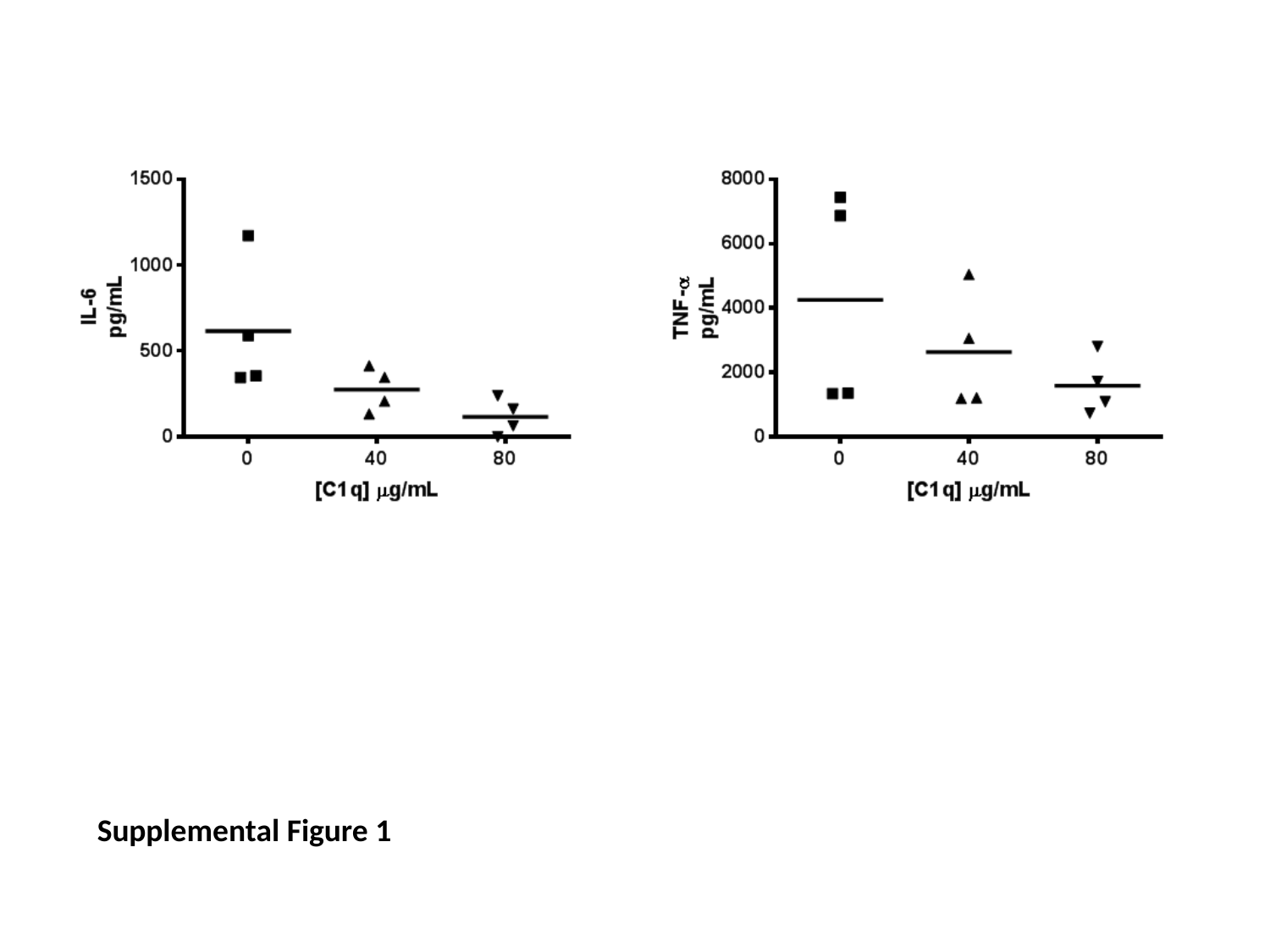

Supplemental Figure 1

Supplement: Additional file 1: Figure S1. — C1q protein inhibits THP-1 macrophage H. pylori stimulated inflammatory cytokine release. THP-1 macrophages were washed and resuspended in serum free medium (Xvivo 10 + L-glut) and pretreated with C1q (0–80 μg/ml) for 30 min before stimulation with H. pylori (MOI 100) for 24 h. Supernatants were collected and TNF-α and IL-6 were measured by specific ELISA. The average and individual values of 2 replicates from n = 2 independent experiments are shown. (PPTX 55 kb) [file 12950_2015_98_MOESM1_ESM.pptx]
